# Enhanced Synthesis of Poly(1,4-butanediol itaconate) via Box–Behnken Design Optimization

**DOI:** 10.3390/polym16192708

**Published:** 2024-09-25

**Authors:** Magdalena Miętus, Mateusz Cegłowski, Tomasz Gołofit, Agnieszka Gadomska-Gajadhur

**Affiliations:** Faculty of Chemistry, Warsaw University of Technology, Noakowskiego 3 Street, 00-664 Warsaw, Poland; magdalena.mietus.dokt@pw.edu.pl (M.M.); mateusz.ceglowski.dokt@pw.edu.pl (M.C.); tomasz.golofit@pw.edu.pl (T.G.)

**Keywords:** Box–Behnken plan, statistical analysis, poly(1,4-butanediol itaconate), tissue engineering

## Abstract

At present, there are too few organ and tissue donors. Due to the needs of the medical market, scientists are seeking new solutions. Those can be found in tissue engineering by synthesizing synthetic cell scaffolds. We have decided to synthesize a potential UV-crosslinked bio-ink for 3D printing, poly(1,4-butanediol itaconate), in response to emerging needs. Diol polyesters are commonly investigated for their use in tissue engineering. However, itaconic acid makes it possible to post-modify the obtained polymer via UV-crosslinking. This work aims to optimize the synthesis of poly(1,4-butanediol itaconate) in the presence of a catalyst, zinc acetate, without using any toxic reactant. The experiments used itaconic acid and 1,4-butanediol using the Box–Behnken mathematical planning method. The input variables were the amount of the catalyst used, as well as the time and temperature of the synthesis. The optimized output variables were the percentage conversion of carboxyl groups, the percentage of unreacted C=C bonds, and the product’s visual and viscosity analysis. The significance of the varying synthesis parameters was determined in each statistical model. The optimum conditions were as follows: amount of catalyst 0.3%_nCOOH_, reaction time 4 h, and temperature 150 °C. The temperature had the most significant impact on the product characteristics, mainly due to side reactions. Experimentally developed models of the polymerization process enable the effective synthesis of a polymer “tailor-made” for a specific application.

## 1. Introduction

**Figure 1:**
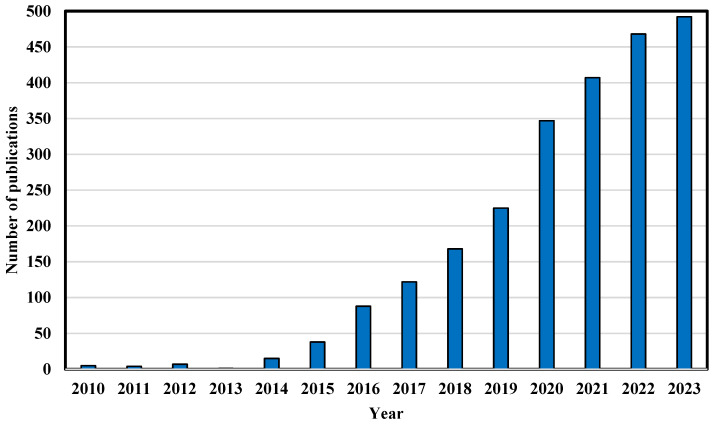
Number of publications with the keyword “bioink” in the years 2010–2023—own elaboration based on data from PubMed page (accessed 20 August 2024).

These days, there is a deficit of tissues and organs for organ transplantations [1]. Therefore, new solutions are being explored [1]. Although still at an early stage of development, one solution is the 3D printing of tissue/organs using bioinks. We call this bioprinting [2]. Bioprinting is becoming increasingly popular, as evidenced by the growing number of publications on 3D printing bioinks (Figure 1).

**Figure 2:**
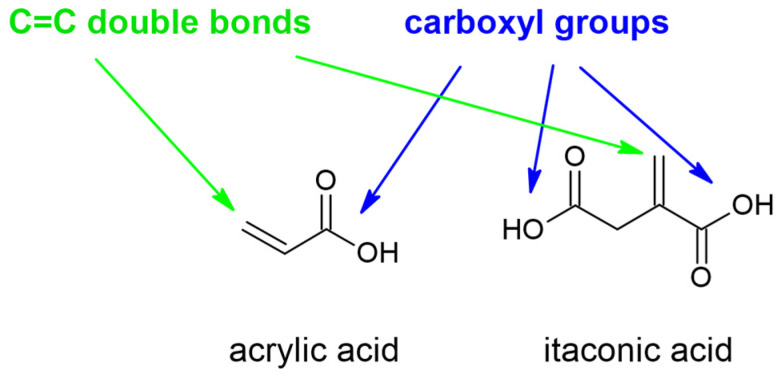
Structure of acrylic acid and itaconic acid.

However, currently used inks have some disadvantages [3,4,5,6]. Most of them contain toxic acrylic compounds in their structure [7]. Those can have a bad influence on the environment [7]. Furthermore, they are produced in petrochemical processes [7]. Because of that, we would like to replace the acrylic compounds with biodegradable itaconic acid (IA), whose structure is very similar to the acrylic acid (Figure 2) [8,9].

Furthermore, the commonly used inks in 3D printing, like gelatin or alginates, are characterized by poor mechanical strength and fragility—they are still unsuitable as tissue/organ substitutes [3,10]. They need to be enriched with other reactants to improve their mechanical properties. Moreover, without modification, the cell’s adhesion to its surface is also poor (for instance, for polylactide or poly(ε-caprolactone) bioinks) [3].

In response to these issues, we have decided to synthesize a potential new bioink—poly(1,4-butanediol itaconate) (PBItc). To the best of our knowledge, there is a lack of articles about synthesizing PBItc without using solvents or radical polymerization reaction inhibitors like MEHQ (4-metoxyphenol) [11,12,13,14]. There are articles about materials synthesis in which IA and 1,4-butanediol (1,4-BD) are only one of the components of more complex materials, like poly(1,4-butanediol-*co*-itaconate-*co*-ricinoleate) or poly(1,4-butanediol/1,3-propanediol/sebacate/itaconate) polyesters [15]. Furthermore, no literature exists about using PBItc as ink in 3D printing. There is a gap to fill in this area, so we have decided to optimize the synthesis of PBItc as a potential bio-ink for 3D printing with a DIW (Direct Ink Writing) method.

**Figure 3:**
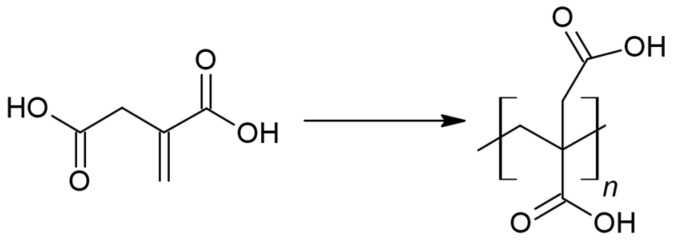
Radical polymerization of itaconic acid.

IA is a biocompatible chemical compound with antimicrobial and anticancerous properties and no toxicity [7,16,17,18,19]. It is a low-cost building block industrially produced through the fermentation of sugars performed by *Aspergillus terreus* fungus [13,15,20]. Furthermore, it is named by the US Department of Energy as one of the 12 top value-added biobased chemicals for industrial purposes [7,9,21]. The presence of a C=C double bond in the structure of the itaconic compound enables the performance of post-polymerization reactions—UV-crosslinking, Michael addition reactions, or Diels–Alder reactions [13,16,22]. Those advantages are essential for IA use in medicine. However, when performing syntheses using itaconic acid, there is a potential for undesirable side reactions. The most relevant undesired reaction in the context of subsequent UV-crosslinking of the product is radical polymerization (Figure 3) [23].

Both IA and the resulting itaconic polymer can undergo this reaction. Because of that, it is essential to perform the synthesis with limited access to light or in the presence of radical polymerization reaction inhibitors like MEHQ, BHT (2,6-di-tert-butyl-4-methyl phenol), or BHA (butylated hydroxyanisole) [24,25,26]. However, those chemical compounds can exhibit narcotic, skin-irritating, or mutagenic effects [27,28]. For these reasons, we have decided to resign from using a radical polymerization reaction inhibitor in the performed syntheses.

**Figure 4:**
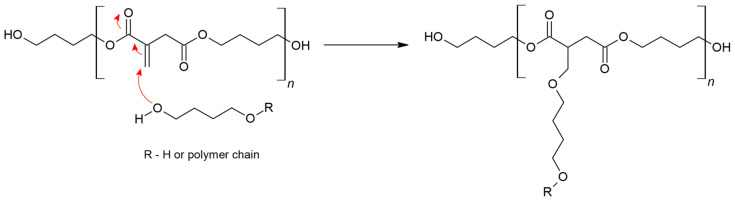
Ordelt reaction of the itaconic compound.

The second critical side reaction in the case of syntheses between itaconic compounds with alcohols is the oxo-Michael addition reaction or, in other words, the Ordelt reaction (Figure 4) [11,23].

Ordelt reaction involves the attachment of a hydroxyl group of a diol to the C=C bond of the itaconic compound [23]. It could lead to the gelation of the reaction system [13]. To minimize the occurrence of the undesired Ordelt reaction, the process should avoid the use of highly acidic Brønsted acids, such as MSA (methanesulfonic acid) or *p*TSA (*p*-toluenesulfonic acid) [1,3]. That is why we have chosen anhydrous zinc acetate, a water-resistant acid [11].

**Figure 5:**
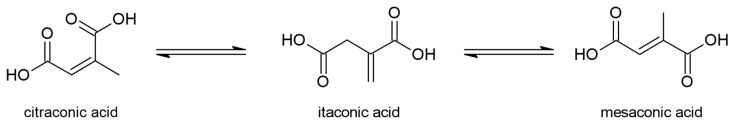
The isomerization of itaconic compound.

Itaconic compounds can isomerise to less reactive mesaconic and citraconic chemicals (Figure 5) [13,23].

The proportion of isomerization reactions increases as the synthesis temperature increases [29,30]. A temperature of 150 °C is considered a limiting temperature, significantly increasing the proportion of isomerization reaction [15,20]. Because of that, it is essential not to synthesize itaconic compounds at higher temperatures, as it can lead to a product with worse mechanical or thermal properties [23]. It will also be more challenging to modify the product, as in the structure of an itaconic compound, the double bond is in the side chain, and the isomers it is in its main chain [23,30,31].

**Figure 6:**
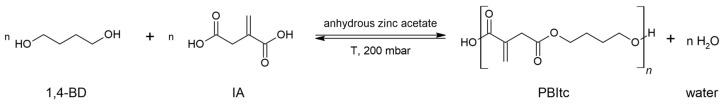
Poly(1,4-butanediol itaconate) synthesis from itaconic acid and 1,4-butanediol.

The second reactant to obtain PBItc (Figure 6) is 1,4-BD.

The compound 1,4-BD is a renewable dihydroxy alcohol found in the human body in trace amounts [32,33]. It is produced in the fermentation process performed by the *E*. *coli* bacteria [34]. The compound 1,4-BD is mainly used as a solvent in industrial processes [33,35]. However, there are more and more articles about its use in the medical field—for medicine encapsulation or as biomaterials [36,37].

The chosen route of synthesizing a new bioink follows the principles of green chemistry [34]. We have examined and discussed in detail the synthesis, in which we chose renewable substrates, resigned from using any solvent or toxic reactants, and utilized a catalyst—anhydrous zinc acetate [34].

## 2. Materials and Methods

### 2.1. PBItc Syntheses Procedure

The syntheses were carried out in a Mettler Toledo MultiMax parallel reactor system (Schwerzenbach, Switzerland) in Hastelloy reactors. The compound 1,4-butanediol (99%, thermo scientific, Kandel, Germany), itaconic acid (≥99%, Sigma-Aldrich, St. Louis, MO, USA), and zinc acetate, anhydrous (99.9+%, Alfa Aesar, Kandel, Germany) were used as supplied without prior preparation.

The reactants 1,4-butanediol (16.37 g, 0.182 mol), itaconic acid (23.63 g, 0.182 mol), and the catalyst, anhydrous zinc acetate (0.2%_nCOOH_ = 0.133 g; 0.3%_nCOOH_ = 0.200 g; 0.4%_nCOOH_ = 0.267 g), were weighed into the metal and non-transparent reactor to minimize the presence of undesired radical crosslinking of itaconic compounds. The molar ratio of the used substrates was 1:1 (1,4-BD:IA). The weight of the used substrates was 40.00 g. Reactors were equipped with mechanical stirrers, temperature sensors, and Dean–Stark apparatuses.

In the first stage of the reaction procedure, the mixture was heated for 15 min to *T* temperature. The temperature was held constant for *t* hours. A total of 30 min after the start of this phase, the reduced pressure was switched on (200 mbar) to shift the equilibrium of the reaction toward product formation (removing the low-molecular-weight product from the reaction environment). Then, the mixture was cooled down to 40 °C for 15 min.

### 2.2. Amount of the Catalyst Used in the Synthesis

The mass of the catalyst used in the performed synthesis reactions was calculated from the following formula:

Mass of the Zn(OAc)_2_ [g] = 2 × *n*_IA_ × (*%n*_COOH_/100) × 183.48 (1)

where

*n*_IA_—the amount of itaconic acid used in the reaction [mol];
*%n*_COOH_—desired catalyst content relative to the molar number of acid groups [%];
183.48—molar mass of the catalyst—zinc acetate.

### 2.3. Acid Number (AN_tit_): Conversion of Carboxyl Groups

Approximately 0.5 g of the PBItc product was weighed and dissolved in 25.00 mL of methanol (Chempur, Piekary Śląskie, Poland). Then, five drops of thymol blue were added. After that, the obtained solution was titrated with a 1 M aqueous NaOH solution (Chempur, Piekary Śląskie, Poland) until the first color change from yellow/orange to blue was observed.

The following formula was used to calculate the acid number:

*AN*_tit_ [mg_KOH_/g_sample_] = ((V − V_0_) × M_NaOH_ × 56.1)/m (2)

where

V—the volume of 1 M NaOH solution used to titrate the investigated sample [cm^3^];
V_0_—the volume of 1 M NaOH solution used for blank titration [cm^3^];
M_NaOH_—the titer of the solution for the titration (1 M);
56.1—the molar mass of KOH [g/mol];
m—the weight of the investigated sample [g].

The final result is the average of three determinations. 

To calculate the conversion of carboxyl groups in the structure of the synthesized product, the following formula was used:

%conv_COOH tit_ = (2 × *n*_IA_ − ((*AN*_tit_/1000)/56.1 × w)/(2 × *n*_IA_)) × 100%

where

*n*_IA_—the amount of itaconic acid used in the synthesis [mol];
w—weight of the substrates in the reaction system [g].

### 2.4. Ester Number (EN_tit_)

Approximately 0.25 g of synthesized PBItc was weighed and dissolved in 15.00 mL of methanol and 20.00 mL of 1 M aqueous NaOH solution. Then, the prepared solution was refluxed for one hour. Next, the mixture was cooled to room temperature, and then five drops of phenolphthalein were added. The excess amount of aqueous NaOH solution was titrated with a 1 M aqueous hydrochloric acid solution (Chempur, Piekary Śląskie, Poland) until the solution was discolored.

To calculate the ester number, the following formula was used:

*EN*_tit_ [mg_KOH_/g_sample_] = (((V_0_ − V) × M_HCl_ × 56.1)/m) − *AN*_tit_ (3)

where

V—the volume of aqueous 1 M HCl solution used to titrate the investigated sample [cm^3^];
V_0_—the volume of aqueous 1 M HCl solution used for blank titration [cm^3^];
56.1—the molar mass of KOH [g/mol];
m—the weight of the investigated sample [g].

The final result is the average of three determinations.

### 2.5. Esterification Degree by Titration (ED_tit_)

The esterification degree was calculated with the use of the following formula:

*ED*_tit_ [%] = *EN*_tit_/(*EN*_tit_ + *AN*_tit_) × 100% (4)

where

*EN*_tit_—ester number from titration;
*AN*_tit_—acid number from titration.

### 2.6. Iodine Number (IN): Percentage of Unreacted C=C Bonds (%_C=C IN_)

Approximately 0.50 g of the investigated sample was weighed into the ground conical flask. Next, 10.00 mL of chloroform (Chempur, Piekary Śląskie, Poland) was added to the sample. The mixture was closed with a cap. After the sample was dissolved, 15.00 mL of Hanus reagent (Chempur, Piekary Śląskie, Poland) was added. The closed flask was thoroughly mixed and placed for 30 min in a dark place. After that, 15.00 mL of 10% KI solution (Chempur, Piekary Śląskie, Poland) and 50 mL of distilled water were added to the mixture. Then, the obtained solution was titrated with 0,1 M sodium thiosulphate (Na_2_S_2_O_3_) solution (Chempur, Piekary Śląskie, Poland) until a bright orange color was observed. Then, approximately 5 mL of starch indicator (Chempur, Piekary Śląskie, Poland) was added to the flask to obtain a dark blue color of the mixture. After that, the solution was titrated with 0.1 M Na_2_S_2_O_3_ solution until it discolored. Two *IN* titrations were performed for each synthesis product.

To calculate the *IN* value, the following formula was used:

*IN* = 1.269 × ((a − b)/c)) (5)

where

a—the volume of sodium thiosulphate solution (0.1 M) used for blank titration [cm^3^];
b—the volume of the sodium thiosulphate solution (0.1 M) used to titrate the sample [cm^3^];
c—the weight of the investigated sample [g].

To calculate the percentage of unreacted C=C bonds in the structure of the PBItc, the following formula was used:

%_C=C IN_ = ((((*IN*/100)/253.81) × w)/*n*_IA_) × 100% (6)

where

253. 81—molar mass of the molecular iodine (I_2_) [g/mol].

### 2.7. Rheological Characterisation

The MCR 301 rheometer (Anton Paar, Graz, Austria) was used to determine the viscosity of the PBItc products. The plate–plate method was applied. The diameter of the moving plate was 25 mm. Every experiment lasted for eight minutes. In the first stage (4 min), the shear rate increased from 0.01 cm^−1^ to 10 cm^−1^. In the second and final stage, the shear rate decreased from 10 cm^−1^ to 0.01 cm^−1^ (4 min). The number of measurement points per decade was 20.

To approximate the measurement curve, the Casson model was used:

(7) $$\tau_{1/2}={\tau^{0}}_{1/2}+{\left(\eta_{p}\times \dot{\gamma }\right)}^{1/2}$$

where

*τ*—shear stress [Pa];
*τ*^0^—shear limit (yield stress) [Pa];
*η*_p_—rheological parameter (plastic viscosity) [Pa × s];
$\dot{\gamma }$—shear rate [s^−1^].

The graphs of the relationship between the square root of the tangential stress and the square root of the shear rate were obtained using the data. For each graph, the regression equation was calculated. The straight line’s directional coefficient was the plastic viscosity’s square root raised to the square. The estimated value was the plastic viscosity.

### 2.8. NMR Analysis

A 400 MHz NMR Spectrometer (Agilent, Santa Clara, CA, USA) was used to record the NMR spectra. Approximately 30.00–60.00 mg of substrates or 130.00–170.00 mg of the synthesis products were weighed into vials on an analytical balance (RADWAG, Radom, Poland). After that, 1 mL of deuterated DMSO (Deuteron GmbH, Kastellaun, Germany) was added. For mathematical calculations, 10 µL of internal standard tert-butanol (t-BuOH) was added to every sample.

The prepared mixtures were closed with a cap and placed on a vibrating shaker (Heidolph 545-10000-00, Schwabach, Germany) to dissolve the vial’s contents. Then, 700 μL of the solution was taken with an automatic pipette (Carl Roth GmbH + Co., Karlsruhe, Germany) and loaded into a glass tube. Afterward, the prepared sample underwent NMR analysis. 

The MestReNova program (version 6.0.2-5457) was used to analyze the NMR spectra.

### 2.9. FTIR Analysis

To perform the FTIR analyses of the samples, approximately 0.5–1.0 g of the substrates and obtained polymers were weighed into the vials using a technical balance (Mettler Toledo, Warsaw, Poland). ALPHA spectrometer (Bruker, Berlin, Germany) was used to perform IR analyses. The measurements used the technique of Attenuated Total Reflectance (ATR). A total of 32 scans in the 400–4000 cm^−1^ range were performed and averaged for each sample.

The OPUS program (version 7.2.139.1294) was used to perform the FTIR analyses.

### 2.10. Gel Permeation Chromatography (GPC) Analysis

Approximately 60.00–70.00 mg of the PBItc was weighed and dissolved in 5 mL of THF for HPLC (Avantor, Gliwice, Poland) for 24 h at 25 °C. Then, the solution was filtered through a syringe filter (pore size = 0.22 µm).

For measurements, an Easy Mate 3000 HPLC chromatograph was used. It was equipped with a Tosoh Bioscience precolumn (cat. 17368) and two Tosoh Bioscience columns (cat. 17368). The used detector was a Shodex RI-101 refractometer detector. A calibration curve was developed using PMMA calibration standards (Easy Vials, Agilent). The measurements were performed at 30 °C with the flow set at 1 mL/min.

### 2.11. Differential Scanning Calorimetry (DSC) Analysis

The Q2000 DSC analyzer (TA Instruments, Eschborn, Germany) was used to perform the DSC analysis.

Every sample weighing approximately 10 mg was weighed into the crucibles closed by lids with holes. Afterward, the chamber was sealed, and the measurement was conducted. The samples were heated twice. The results of the polycondensation reaction were shown in the first heating curve. On the second heating curve, the fully reacted products were investigated. DSC analyses were conducted in the nitrogen flow (50 mL/min).

The DSC procedure was as follows. In the first step, the sample was cooled to −90 °C. Then, it was heated to 250 °C (10 °C/min step). In the next step, the sample was cooled to −100 °C. In the final stage, the sample was heated again to 250 °C.

DSC thermograms were analyzed using TA Instruments Universal Analysis 2000 software.

The glass transition temperature was determined as a midpoint temperature. The cold crystallization temperature was defined as a peak temperature.

### 2.12. Thermogravimetry (TG) Analysis

An SDT Q600 analyzer (TA Instruments, Eschborn, Germany) performed TG analysis. The analysis was performed on the samples weighing 10 mg. The weight loss of the samples was analyzed using the temperature range from room temperature to 500 °C (10 °C/min step). TG analyses were conducted in the nitrogen flow (100 mL/min).

### 2.13. Viscosity-Visual-Utility Analysis (VVU)

The numerical interval scale characterized every synthesis product from the mathematical model for its specified properties (Table 1). The numerical interval scale was converted into a percentage scale. The highest number of possible points, 20, was defined as 100%, and the lowest number of possible points, 6, was described as 0%.

### 2.14. Solubility Analysis

Approximately 1.0 g samples of the investigated PBItc samples were weighed into the vials using a technical balance (Mettler Toledo, Warsaw, Poland). Then, around 10.0 g of the solvent was added to each vial. The solubility of the PBItc was confirmed in the following solvents: *n*-hexane (POCH, Gliwice, Poland), toluene (Chempur, Piekary Śląskie, Poland), diethyl ether (Chempur, Piekary Śląskie, Poland), ethyl alcohol (POCH, Gliwice, Poland), dichloromethane (POCH, Gliwice, Poland), THF (POCH, Gliwice, Poland), chloroform (Chempur, Piekary Śląskie, Poland), ethyl acetate (POCH, Gliwice, Poland), 1,4-dioxane (POCH, Gliwice, Poland), methanol (Chempur, Piekary Śląskie, Poland), acetone (POCH, Gliwice, Poland) acetonitrile (POCH, Gliwice, Poland), 1-butanol (POCH, Gliwice, Poland), DMF (POCH, Gliwice, Poland), DMSO (Chempur, Piekary Śląskie, Poland), and distilled water. The prepared samples were agitated for 24 h on a vibrating shaker (Heidolph 545-10000-00, Schwabach, Germany). After that time, the solubility of PBItc in a given solvent was determined.

## 3. Results and Discussion

### 3.1. FTIR and NMR Analysis

**Figure 7:**
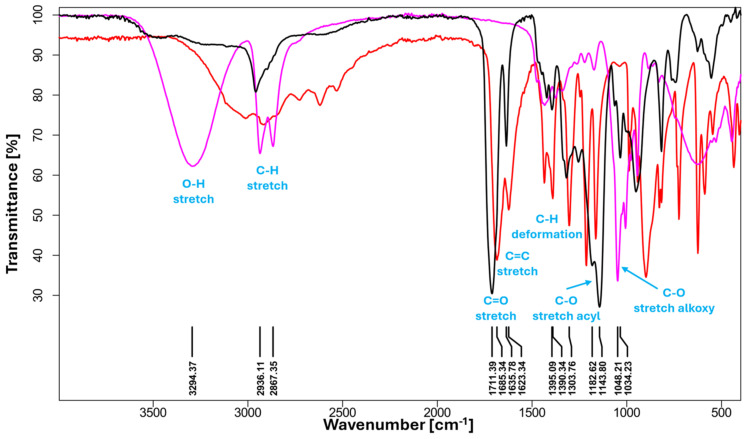
FTIR spectra of poly(1,4-butanediol itaconate) (**black line**), itaconic acid (**red line**), and 1,4-butanediol (**pink line**).

The structure of PBItc was confirmed by the Fourier Transform Infrared Spectroscopy (FTIR) (Figure 7). The FTIR analysis was compared and confirmed with to-date literature reports [11].

The PBItc spectra show a small intensity band in the 3550–3200 cm^−1^ range, corresponding to the O-H bond stretching vibrations. Because of its intensity, it can be concluded that a small proportion of the unreacted 1,4-butanediol and itaconic acid can be observed in the final PBItc product. The bands in the range of 2940–2850 cm^−1^ correspond to the stretching vibrations of the methylene groups vibrations. The most essential bands observed are below 2000 cm^−1^. Firstly, stretching vibrations of the carbonyl groups correspond to the *α* and *β*-unsaturated esters (1730–1715 cm^−1^). Then, stretching vibration of the C=C bond (range of 1640–1620 cm^−1^). The intensity of the signals from the product is lower than from the IA, which means that the undesired reactions involving the C=C bond take place. In the 1200–1000 cm^−1^ range, signals confirm the presence of polyester in the reaction system. Those are the C-O stretching vibrations of the acyl groups (range of 1190–1140 cm^−1^) and of the acyl groups (range of 1050–1030 cm^−1^).

**Figure 8:**
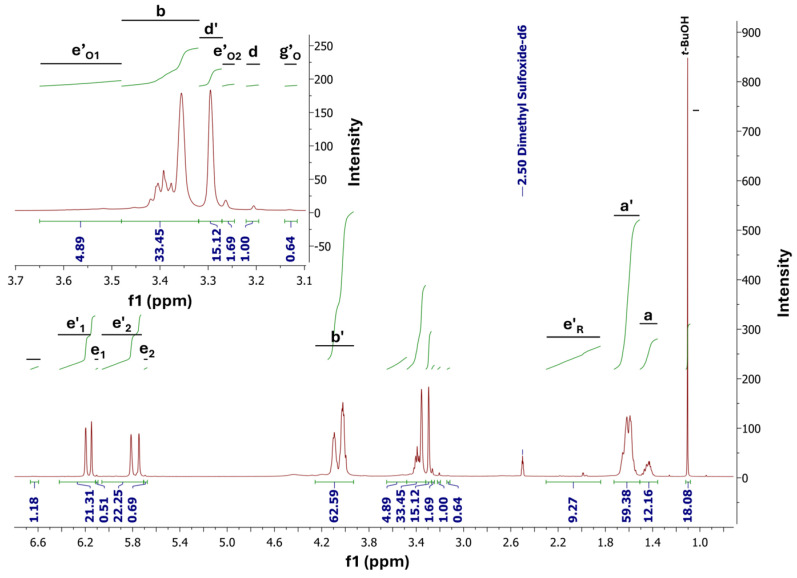
^1^H NMR spectra of poly(1,4-butanediol itaconate) synthesized in the presence of anhydrous zinc acetate.

**Figure 9:**
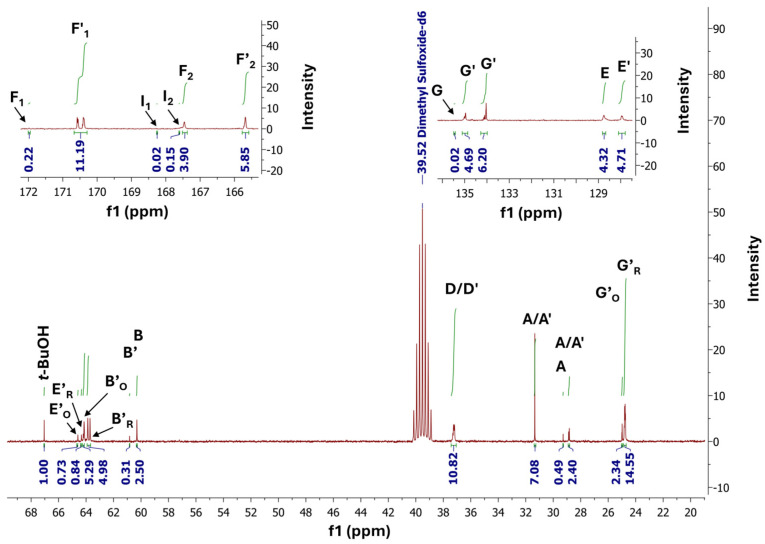
^13^C NMR-NOE spectra of poly(1,4-butanediol itaconate) synthesized with anhydrous zinc acetate.

The ^1^H Nuclear Magnetic Resonance (^1^H NMR) (Figure 8) and ^13^C-NOE Nuclear Magnetic Resonance (^13^C NMR-NOE) (Figure 9) spectra were used to confirm the PBItc structure and to perform relevant calculations (view the Equations Section in the Supplementary Information). The assignment of protons and carbon atoms to the signals on NMR spectra can be seen in Figure S1. The NMR analyses were compared and confirmed with to-date literature reports [12,15,40].

The signals which correspond to the presence of the desired PBItc product are a′, b′, d′, e′_1_, and e′_2_. Because of the existence of undesired side reactions, the protons that correspond to the Ordelt reaction (signals e′_O1_, e′_O2_, and g′_O_) undergo an isomerization reaction to form a mesaconic compound (h), and radical polymerization reaction (e′_R_) can also be seen. As the reaction has not been fully completed, there are also signals from the unreacted substrates—IA (d, e_1_, and e_2_) and 1,4-BD (a, b).

The ^13^C NMR-NOE spectra confirm the presence of the PBItc in the reaction system. There are signals from the carbonyl carbons (173–164 ppm) which refer to the main product (F′_1_ and F′_2_ signals), unreacted IA (F_1_ and F_2_), and undesired mesaconic compound (I_1_ and I_2_). In the 140–125 ppm, 70–60 ppm, and 35–20 ppm ranges, most peaks correspond to undesired side reaction products and unreacted substrates.

### 3.2. Statistical Analysis

The Box–Behnken plan was used to optimize the synthesis of PBItc in the presence of anhydrous zinc acetate. The chosen matrix consists of fifteen experiments, three of which have been performed under the same conditions to verify the repeatability of the results and the experimenter’s conscientiousness.

The chosen input (*x*) and output (*y*) variables for optimizing the PBItc synthesis are shown below.

- *x*_1_—amount of the used catalyst (%_nCOOH_);
- *x*_2_—time of the PBItc synthesis (t) [h];
- *x*_3_—temperature of the PBItc synthesis (T), [°C];
- *y*_1_—percentage conversion of carboxyl groups -COOH (*conv*_COOH tit_) (calculated from the *AN*_tit_);
- *y*_2_—percentage of unreacted unsaturated C=C double bonds (%_C=C 13C NMR_) (calculated from the ^13^C NMR-NOE spectra analysis);
- *y*_3_—Viscosity-Visual-Utility analysis (%_VVU_).

Compared with the literature reports, we have decided to calculate the amount of catalyst relative to the amount of carboxyl groups in the structure of IA as they primarily react with the catalyst [11,41].

**Figure 10:**
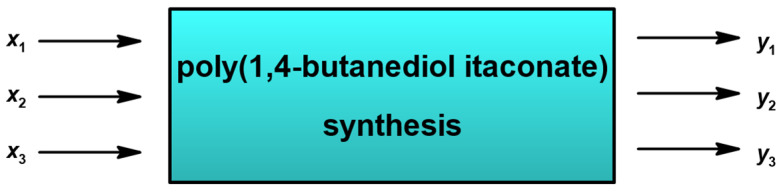
“Black box” of the poly(1,4-butanediol itaconate) synthesis process.

The discussed process of PBItc synthesis is presented as a “black box” in Figure 10. The coded values of the chosen input variables have been summarised in Table S1.

The experimentally-calculated and model-calculated values of the investigated output variables are presented in Table 2. Figure S5 shows the consistency of the products obtained in the performed experiments. Other important calculated parameters for the obtained products have been shown in Table S2.

The *t*-Student test was used to define if the regression equation coefficients are significant (|*t*_calculated_| > *t*_critical_) for the investigated output variables (Table S4). The probability level value (*p*-value) was set to 5% (*t*_critical_ = 2.57). The Analysis of Variance (ANOVA) results are graphically presented on the Pareto Charts (Figures S2–S4). The model adequacy tests for the output variables are shown in Table S5.

For the *y*_1_ output variable, the significant coefficients are as follows: *x*_3_ (|*t*_calculated_| = 64.6), *x*_2_ (|*t*_calculated_| = 14.6), *x*_3_^2^ (|*t*_calculated_| = 7.05), linear relationship between *x*_1_ and *x*_3_ (|*t*_calculated_| = 5.84), *x*_1_ (|*t*_calculated_| = 5.43), *x*_2_^2^ (|*t*_calculated_| = 4.91), and linear relationship between *x*_2_ and *x*_3_ (|*t*_calculated_| = 4.66). Comparing the R^2^ coefficient of determination value, whether including an insignificant coefficient would increase the R^2^ value has been checked. Including all irrelevant variables in the regression equation slightly increases the R^2^ value (from 0.9981 to 0.9989). Because of that, all of the coefficients have been included in the *y*_1_ regression equation (Equation (S9)).

The equation which describes the percentage conversion of carboxyl groups -COOH (*y*_1_) (with the inclusion of only relevant coefficients) is as follows:

*y*_1_ = 56.5 + 1.07 × *x*_1_ + 2.87 × *x*_2_ + 12.7 × *x*_3_ + 1.63 × *x*_1_ × *x*_3_ + 1.30 × *x*_2_ × *x*_3_ − 1.43 × *x*_2_^2^ + 2.04 × *x*_3_^2^

The *F*-Snedecor test was used to determine the adequacy of the regression equations. The regression equation is adequate if *F*_calculated_ < *F*_critical_. For *y*_1_ output variable *F*_calculated_ = 1.40, there is no evidence to reject the hypothesis that the above equation is sufficient. 

**Figure 11:**
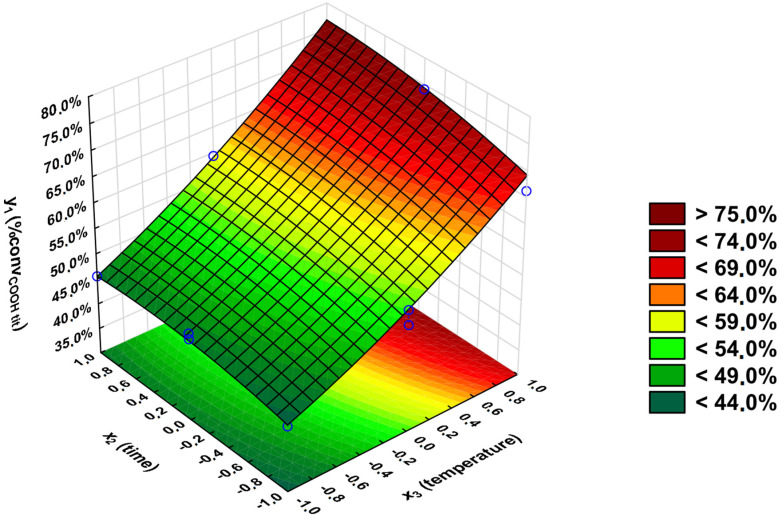
Dependence of the percentage conversion of carboxyl groups -COOH on the reaction’s temperature (*x*_3_) and time (*x*_2_); *x*_1_ = 1.

The graphical representation of the regression equation for output variable *y*_1_ is presented in Figure 11.

The experimental and calculated values of the *y*_1_ output variable differ only from −0.5 to 0.6 percentage points (Table 2). This means that the applied model fits very well. The highest conversion of carboxyl functional groups can be obtained at the highest investigated temperature. The lower the reaction’s temperature, the smaller the *y*_1_ value. As shown in Figure 9, temperature plays a more crucial role in obtaining the high *y*_1_ value than the reaction’s time. This is confirmed by the ANOVA analysis, which shows that the coefficient from the *x*_3_ input variable is more significant than that from the *x*_2_ input variable.

**Figure 12:**
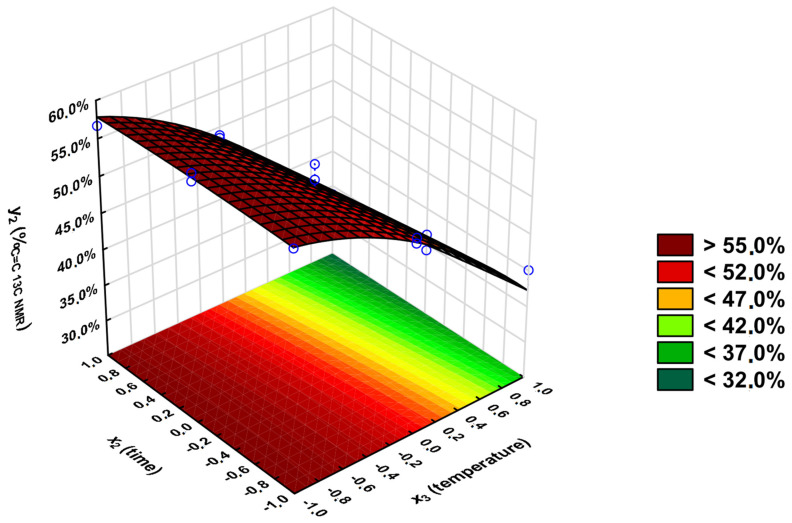
Dependence of the percentage of unreacted unsaturated C=C double bonds on the reaction’s temperature (*x*_3_) and time (*x*_2_); *x*_1_ = 1.

For the *y*_2_ variable, the input variables that are significant are *x*_3_ (|*t*_calculated_| = 18.8), *x*_3_^2^ (|*t*_calculated_| = 4.30), and *x*_2_ (|*t*_calculated_| = 2.69). As the inclusion of all coefficients in the regression equation (including non-significant ones) increases the R^2^ value (from 0.97 to 0.99), they were included in the calculations to obtain the response surface graph (Figure 12).

The equation below describes the percentage of unreacted unsaturated C=C double bonds (*y*_2_) (including only relevant coefficients). The equation with all of the coefficients is shown as Equation (S10).

*y*_2_ = 50.9 − 10.7 × *x*_3_ − 1.53 × *x*_2_ − 3.61 × *x*_3_^2^

For the *y*_2_ output variable, *F*_calculated_ = 0.31 means there is no evidence to reject the hypothesis that the equation presented above is sufficient.

The experimental and calculated values of the *y*_2_ output variable differ by ±0.9 percentage points (Table 2), which concludes that the applied model is very well-fitted. Once again, the temperature plays the most crucial role in obtaining the highest percentage of unreacted C=C double bonds (>55.0%). To obtain the highest *y*_2_ value, performing the syntheses at a lower temperature is crucial. The *x*_2_ input variable plays a more significant role in higher temperatures (150 °C).

It can be concluded from the Pareto chart analysis (Figure S4) that only the *x*_3_ input variable (|*t*_calculated_| = 2.65) significantly affects the output variable *y*_3_. The R^2^ value, including only the significant variable, is 0.25. Therefore, all the insignificant factors were included (Equation (S11)), and R^2^ rose to 0.83.

*y*_3_ = 76.7 + 8.75 × *x*_3_

**Figure 13:**
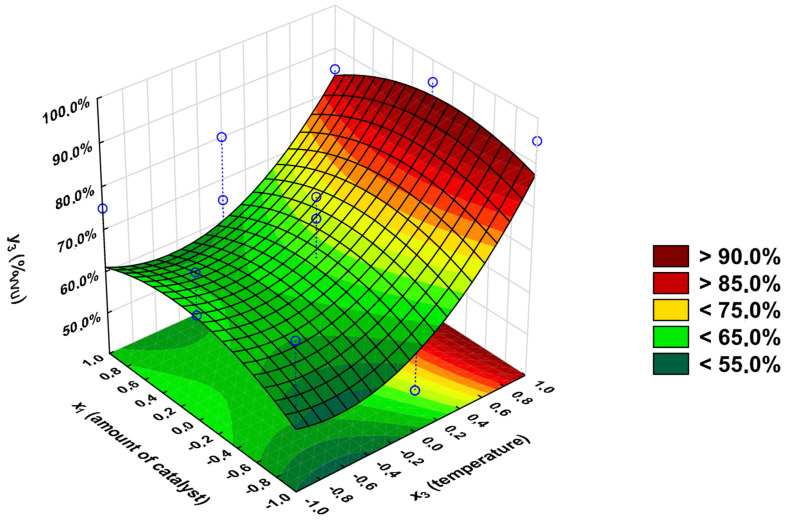
Dependence of the Viscosity-Visual-Utility analysis on the reaction’s temperature (*x*_3_) and amount of catalyst (*x*_1_); *x*_2_ = 1.

Based on the *F*-test, it has been concluded that the used model is adequate (*F*_calculated_ = 16.75). The graphical representation of the regression equation for output variable *y*_1_ is presented in Figure 13.

In the case of the *y*_3_ output variable, its calculated and experimental values differ from −8.8 to 8.7 percentage points, which shows a perfect fit for the applied model. However, it must be noted that the significant change in the R^2^ coefficient of determinations value means that the non-significant variable also influences the value of the *y*_3_ variable and probably other parameters that were not considered in the analysis. The change in the amount of the catalyst slightly changes the *y*_3_ value when the reaction is performed at a constant temperature. To obtain the highest %_VVU_ value, the reaction should be performed at the highest temperature (150 °C) in the presence of 0.3%_nCOOH_ catalyst (%_VVU_ > 90.0%).

### 3.3. Optimal Conditions Synthesis

We have used the least square method available in Statistica’s response utility profile function software to find the optimal conditions of the synthesis of PBItc. For this, we have used the utility profile function software (Figure S6) and programmed the output variables as variables with low, medium, and high utility (Table S3). We have determined that the highest values of the investigated output variables (with consideration of the utility value of the model) can be obtained under the following conditions: the amount of catalyst = 0.3%_nCOOH_ (*x*_1_), reaction time = 4 h (*x*_2_), and reaction temperature = 150 °C (*x*_3_). The consistency of the obtained product is shown in Figure S6. The product was a yellowish resin, similar to the poly(1,3-propanediol itaconate) obtained in the presence of zinc acetate [11].

The experiment was carried out under the assumed conditions, summarising the analysis results in Table 3.

The calculated value of the investigated *y*_1_ variable is higher than that of the profile of approximated values used. However, the difference is only 0.4 percentage points. The reason for this may be the experimenter’s method of titration. The difference between calculated and experimental *y*_2_ and *y*_3_ for the remaining output variables is consecutively 1.5 and 2.7 percentage points. It indicates a good fit of the statistical models to the reality.

In comparison with the literature reports, in the performed synthesis, the reaction time was shorter (4 h in comparison with 6 h), and the temperature was lower (150 °C in contrast with 160 °C) [14,42]. It shows that it is possible to obtain a product with desired characteristics in milder conditions and without the use of toxic reactants—MEHQ or BHT [11,14]. However, it should also be noted that, in the article [12,14,42], the authors have used a different catalyst, *p*TSA, which is not recommended for synthesizing itaconic compounds.

The GPC analysis results show that the synthesized product’s molecular weight was, successively, *M*_n_ = 1374 and *M*_w_ = 1487 (*DI* (Dispersity Index) = 1.08). The obtained weight is one of the highest from all fifteen experiments. It is also higher than in literature reports of similar compounds (*M*_n_ consecutively: 831 [36], 580 [40]). The obtained molecular weight is lower than the polyesters synthesized by adding other acids or diols [15,41]. For this reason, in the future, we are considering testing the addition of different compositions, like itaconic acid–succinic anhydride–diol.

Even though using excess acid should result in a product with a lower proportion of Ordelt reaction, to achieve a product with the highest possible molar mass, we have decided to use the 1:1 molar ratio [9]. The proportion of Ordelt’s reaction was *%Ord* = 14.1%, one of the lowest obtained (11.0–22.8%). Furthermore, the proportion of undesirable isomerization reaction was also low, *%Iz*_Mes_ ≤ 2%. For comparison, in the literature, the proportion of less reactive mesaconic isomers can exceed 30% for PGI (poly(glycerol itaconate) synthesis [40].

**Figure 14:**
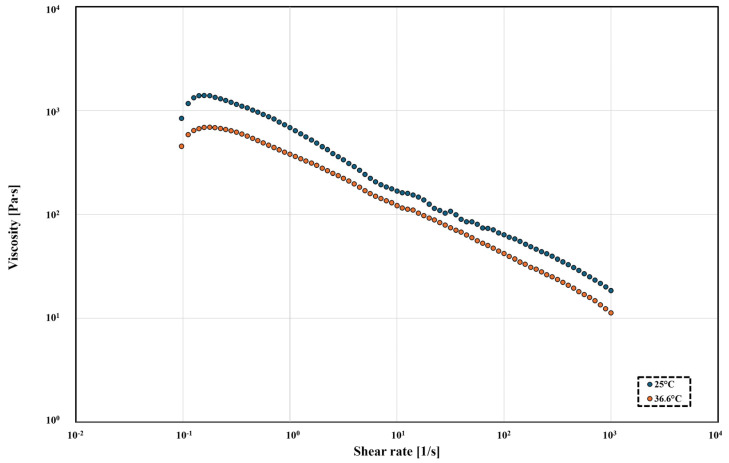
Continuous flow tests of poly(1,4-butanediol itaconate) at shear rates equivalent to those experienced during printing in two temperatures: 25 °C (blue) and 36.6 °C (orange).

The obtained product was characterized by its rheological, thermal, and solubility properties. The change in viscosity of the obtained PBItc material has been shown in Figure 14.

At a temperature of 25 °C, the plastic viscosity of PBItc is 71.7 Pa∙s, and, at 36.6 °C, it is 30.1 Pa∙s. The obtained poly(1,4-butanediol itaconate) is a fluid diluted by shearing, as, with the increase in shear rate, the viscosity decreases. It is a desired property for a bio-ink as it will facilitate the printing process [10,43]. The obtained PBItc viscosity is lower than 100 Pa∙s, which (according to the literature reports) is appropriate for 3D printing with the DIW method [38,39].

**Figure 15:**
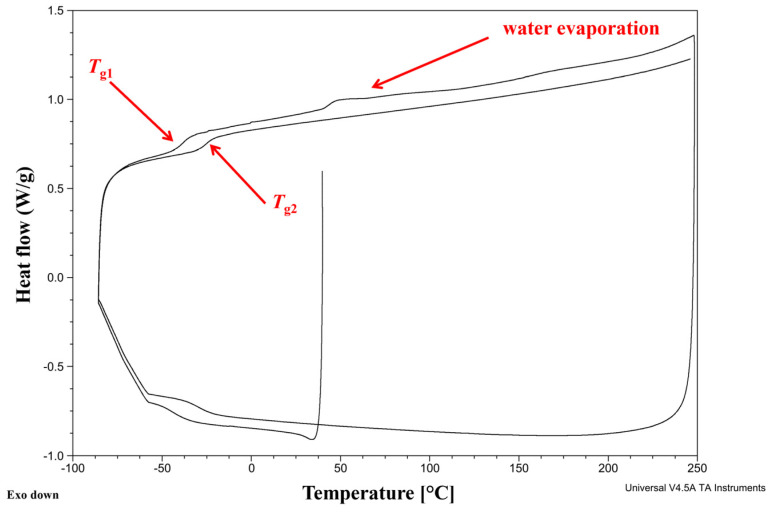
DSC analysis of the optimal poly (1,4-butanediol itaconate) synthesis product in the presence of anhydrous zinc acetate (where *T*_g1_—glass transition temperature during first heating; *T*_g2_—glass transition temperature during second heating).

The Differential Scanning Calorimetry (DSC) analysis (Figure 15) shows two characteristic temperatures (Table 4).

We have calculated the statistical heat resistance of PBItc, which is 130.3 °C. There are no visible bulges corresponding to the evaporation of water from the reaction system. This means applying reduced pressure in the reaction system helped remove it from the resulting product. The appointed glass temperatures are higher than those reported in the literature for PBItc (ranging from −44.8 °C to −39.6 °C) [40]. These can result in a product with better mechanical strength or stability, which are crucial for a medical use material.

**Figure 16:**
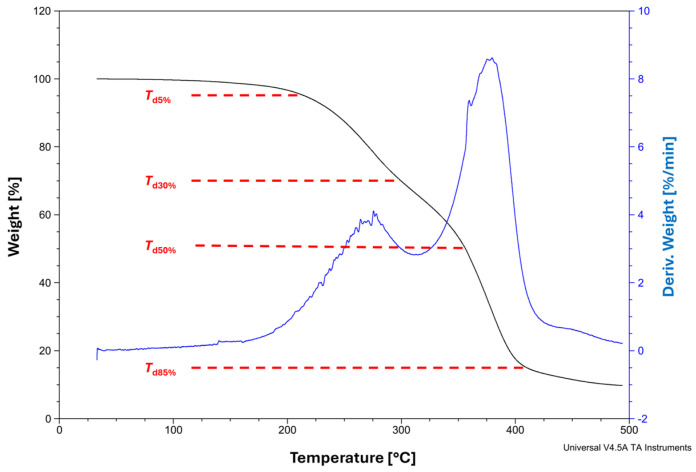
TG (**black**) and DTG (**blue**) analysis of the optimal poly(1,4-butanediol itaconate) synthesis product in the presence of anhydrous zinc acetate.

The Thermogravimetry (TG) analysis (Figure 16) shows that the obtained PBItc is characterized by two-step decomposition as two inflection points can be seen on the DTG analysis (blue curve). The first clearly visible mass loss has its peak at the temperature of around 250 °C. Then, the product shows stability. At around 400 °C, a second mass loss takes place. It is associated with the remaining chain fragmentation and dehydrogenation [44,45].

**Figure 17:**
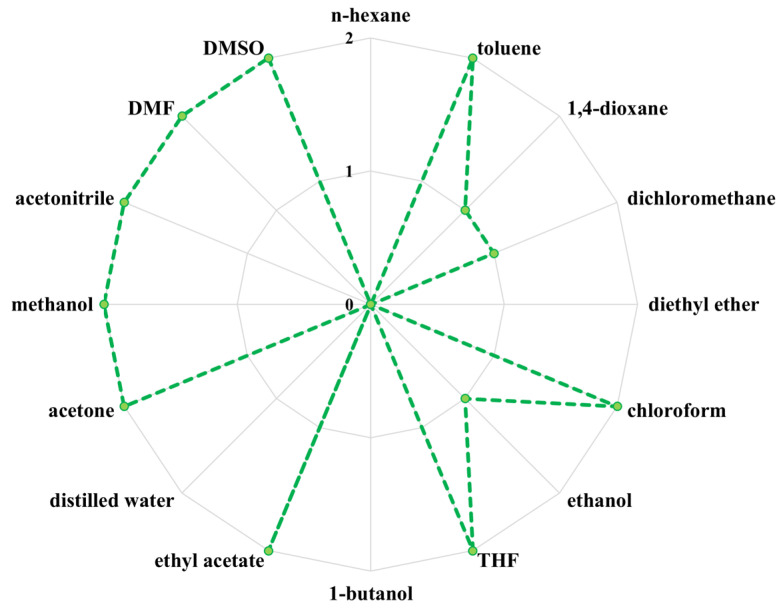
Solubility chart of the optimal poly(1,4-butanediol itaconate) synthesis product in the presence of anhydrous zinc acetate (where: 0—no solubility, 1—partial solubility, 2—complete solubility). The solvents are arranged with increasing dipole moment (from *n*-hexane to DMSO).

For future analyses, the product synthesized in the optimal conditions was also investigated for its solubility properties in commonly used solvents (Figure 17).

The obtained PBItc shows excellent solubility in more than half of the investigated solvents. It provides a good prognosis before performing analyses on crosslinked films, for instance, hydrolytic degradation, swelling analysis, or gel content. It also confirms that the obtained polymer chains are flexible enough that the solvents’ molecules can easily penetrate the structure of the polymer chains and separate them.

## 4. Conclusions

In this study, we synthesized and characterized poly(1,4-butanediol itaconate) to examine it further as a potential bio-ink in 3D printing. We have drawn attention to the challenges associated with synthesizing itaconic polyesters—radical polymerization reaction, Ordelt reaction, and isomerization reaction. We have used the Box–Behnken mathematical model to optimize the synthesis of poly(1,4-butanediol itaconate) in the presence of anhydrous zinc acetate to minimize the presence of these undesired reactions. The results show that it is possible to synthesize a promising product fully from renewable substrates without using any toxic or petrochemical reactants. We were also able to minimize the presence of undesired isomerization reactions. However, it is hard to significantly minimize the proportion of other undesired reactions that lead to the content reduction of unreacted C=C bonds required for UV-crosslinking. The obtained results demonstrated that poly(1,4-butanediol itaconate) must be synthesized in the presence of 0.3%_nCOOH_ anhydrous zinc acetate for 4 h at 150 °C. It was observed that temperature and reaction time have the highest influence on the properties of the synthesis product. As for now, the obtained PBItc has the potential to be used as a polymeric bio-ink. Because of that, further investigation is now ongoing in our lab. Shortly, we will perform the UV-crosslinking of our synthesized products and characterize them deeply (mechanical, wetting angle analysis, degradation, cytotoxicity tests) to confirm if the obtained poly(1,4-butanediol itaconate) is an appropriate material for 3D bioprinting. We want to perform UV-crosslinking of the obtained poly(1,4-butanediol itaconate) to examine if adding the photoinitiator is required for the system. We will also perform printability tests and prepare a broad comparison between PBItc and other commonly examined materials.

## Figures and Tables

**Table 1:** Viscosity-Visual-Utility evaluation for the obtained poly(1,4-butanediol itaconate) products.

Structure of the Obtained PBItc		Consistency of the Obtained PBItc		Transparency of the Obtained PBItc		Ability to Spread the PBItc Sample on the Table in *T*_room_ *		^a^ Viscosity at (a) T_room_ and (b) T = 36.6 °C	
1	Hard and brittle	1	Wax	1	None	1	Yes	1	η < 10 or η > 1000
2	Incompressible and sticky	2	Wax/Resin	2	Partial	2	Partial	2	500 < η < 1000
3	Compressible and sticky	3	Resin	3	Full	3	No	3	100 < η < 500
								4	10 < η < 100

* *T*_room_—room temperature. ^a^ The viscosity limits were chosen after analyzing literature reports about DIW inks [38,39].

**Table 2:** Experimental matrix and the calculated results of the Box–Behnken plan.

No.	Coded Variable			*conv*_COOH tit_ [%]			%_C=C 13C NMR_ [%]			%_VVU_ [%]		
	*x*_1_	*x*_2_	*x*_3_	Exp. *	Calc. *	Diff. *	Exp.	Calc.	Diff.	Exp.	Calc.	Diff.
**1**	**−1**	**−1**	**0**	51.0	51.2	−0.2	51.6	52.0	−0.3	50.0	55.0	−5.0
**2**	**1**	**−1**	**0**	53.8	54.1	−0.2	50.9	51.0	−0.2	80.0	71.3	8.7
**3**	**−1**	**1**	**0**	57.9	57.7	0.2	48.5	48.3	0.2	50.0	58.8	−8.8
**4**	**1**	**1**	**0**	59.3	59.1	0.2	48.9	48.5	0.3	65.0	60.0	5.0
**5**	**−1**	**0**	**−1**	47.1	46.8	0.3	57.5	56.8	0.8	75.0	66.9	8.1
**6**	**1**	**0**	**−1**	46.0	45.7	0.3	58.6	58.0	0.6	75.0	80.6	−5.6
**7**	**−1**	**0**	**1**	68.7	69.0	−0.3	36.3	36.9	−0.6	95.0	89.4	5.6
**8**	**1**	**0**	**1**	74.1	74.4	−0.3	34.2	34.9	−0.8	85.0	93.1	−8.1
**9**	**0**	**−1**	**−1**	42.8	42.9	−0.1	57.5	57.9	−0.4	75.0	78.1	−3.1
**10**	**0**	**1**	**−1**	45.5	46.0	−0.5	56.7	57.6	−0.9	65.0	64.4	0.6
**11**	**0**	**−1**	**1**	66.2	65.7	0.5	40.1	39.2	0.9	85.0	85.6	−0.6
**12**	**0**	**1**	**1**	74.1	74.0	0.1	33.7	33.3	0.4	95.0	91.9	3.1
**13**	**0**	**0**	**0**	56.3	56.5	−0.3	53.0	50.9	2.1	80.0	76.7	3.3
**14**	**0**	**0**	**0**	57.1	56.5	0.6	48.8	50.9	−2.1	75.0	76.7	−1.7
**15**	**0**	**0**	**0**	56.2	56.5	−0.3	50.9	50.9	0.0	75.0	76.7	−1.7

* Exp.—Experimental, Calc.—Calculated, Diff.—Difference.

**Table 3:** Calculated and experimental results of the output variables for the product obtained in the optimal conditions.

Results	*conv*_COOH tit_ [%]	%_C=C 13C NMR_ [%]	%_VVU_ [%]
**Calculated**	71.3	36.5	97.7
**Experimental**	71.7	35.0	95.0

**Table 4:** Thermal analysis of poly(1,4-butanediol itaconate).

**DSC Analysis**			
***T*_g1_** **[°C]**		***T*_g2_** **[°C]**	
−38.1		−24.9	
**TG Analysis**			
***T*_d5%_** **[°C]**	***T*_d30%_** **[°C]**	***T*_d50%_** **[°C]**	***T*_d85%_** **[°C]**
215.2	299.6	356.3	410.0

## Data Availability

Data are contained within the article.

## Supplementary Materials

The following supporting information can be downloaded at: https://www.mdpi.com/article/10.3390/polym16192708/s1, Figure S1: The proton and carbon atoms assignment to the corresponding signals on the ^1^H NMR and ^13^C spectra; Figure S2: Pareto Chart of Standardize Effects for the *conv*_COOH tit_ (*y*_1_) variable (the red line refers to the limit, beyond which the coefficient of the regression equation becomes significant); Figure S3: Pareto Chart of Standardize Effects for the %_C=C 13C NMR_ (*y*_2_) variable; Figure S4: Pareto Chart of Standardize Effects for the %_VVU_ (*y*_3_) variable; Figure S5: Consistency of the products obtained in the optimization process; Figure S6: Consistency of the optimal product; Figure S7: Profile of approximated values of input variables and utility of the used mathematical model; Table S1: Coded input variables in the Box–Behnken plan for the PBItc synthesis optimization; Table S2: Number *(M*_n_) and weight (*M*_w_) average molar mass and dispersity index (*DI*) calculated from GPC analyses; isomerization to mesaconic acid (*%Iz*_Mes_), number average molar weight *(M*_n_), and esterification degree (*ED*_NMR_) calculated from ^1^H NMR analyses; percentage conversion of carboxyl groups -COOH calculated from ^13^C NMR analysis (*conv*_COOH 13C NMR_); esterification degree (*ED*_tit_), percentage of unreacted unsaturated C=C bonds (%_C=C IN_) calculated from titration analyses and viscosity for the products from the Box–Behnken plan; Table S3: Values of output variables to generate the response utility profile; Table S4: Significance test of regression equation coefficients for the investigated output variable; Table S5: Model adequacy test for the investigated output variables.

## Abbreviations

^1^H NMR—^1^H Nuclear Magnetic Resonance; ^13^C NMR-NOE—^13^C-NOE Nulcear Magnetic Resonance; 1,4-BD—1,4-butanediol; ANOVA—Analysis of Variance; IA—itaconic acid; BHA—butylated hydroxyanisole; BHT—2,6-di-tert-butyl-4-methyl phenol; DI—Dispersity Index; DIW—Direct Ink Writing; DSC—Differential Scanning Calorimetry; FTIR—Fourier Transform Infrared Spectroscopy; MEHQ—4-metoxyphenol; MSA—methanesulfonic acid; PBItc—poly(1,4-butanediol itaconate); PGI—poly(glycerol itaconate); *p*TSA—p-toluenesulfonic acid; TG—Thermogravimetry.
