# Supplementary material for: Enhanced Synthesis of Poly(1,4-butanediol itaconate) via Box–Behnken Design Optimization
Source: Polymers (Basel). 2024 Sep 25;16(19):2708. doi: 10.3390/polym16192708 (PMC11479127; doi:10.3390/polym16192708)
Supplement: Supplementary file 1 [file polymers-16-02708-s001.zip › polymers-3205571-supplementary.pdf]

Enhanced Synthesis of Poly(1,4-butanediol itaconate) via Box–Behnken Design Optimization

Magdalena Miętus, Mateusz Cegłowski, Tomasz Gołofit and Agnieszka Gadomska-Gajadhur *

Faculty of Chemistry, Warsaw University of Technology, Noakowskiego 3 Street, 00-664 Warsaw, Poland; magdalena.mietus.dokt@pw.edu.pl (M.M.); mateusz.ceglowski.dokt@pw.edu.pl (M.C.); tomasz.golofit@pw.edu.pl (T.G.)
* Correspondence: agnieszka.gajadhur@pw.edu.pl

Keywords Box–Behnken plan; statistical analysis; poly(1,4-butanediol itaconate); tissue engineering

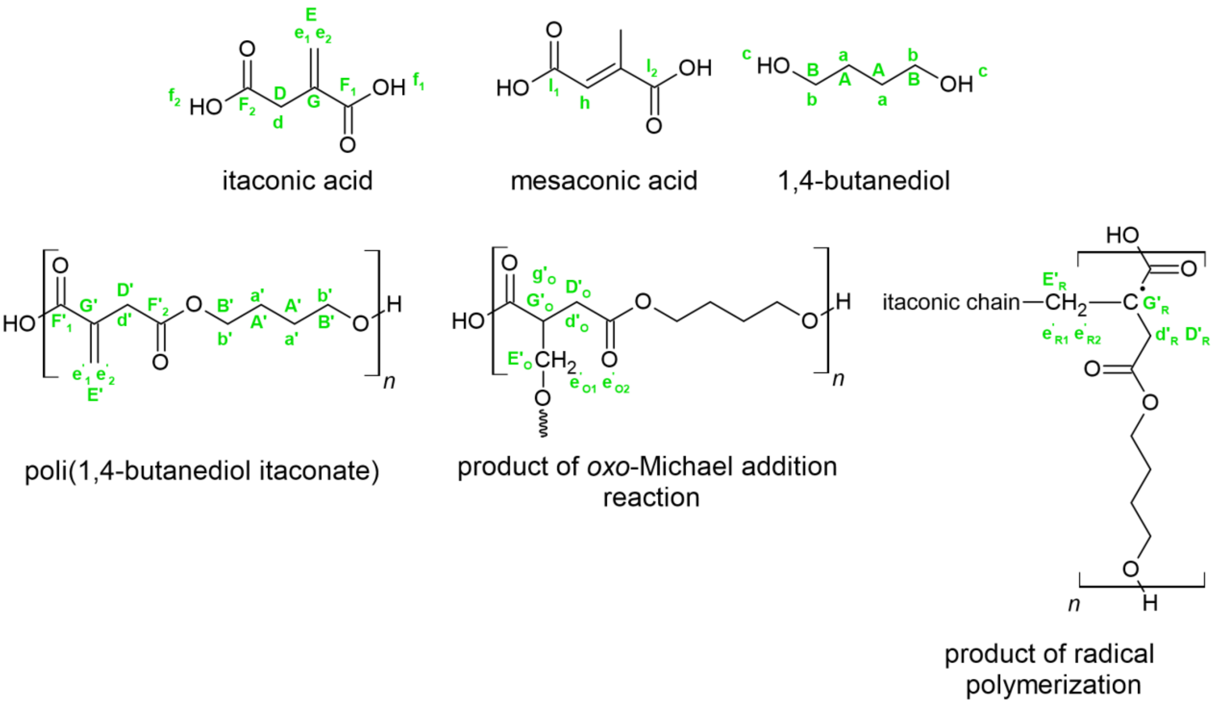

Figure S1. The proton and carbon atoms assignment to the corresponding signals on the ^{1}H NMR and ^{13}C spectra.

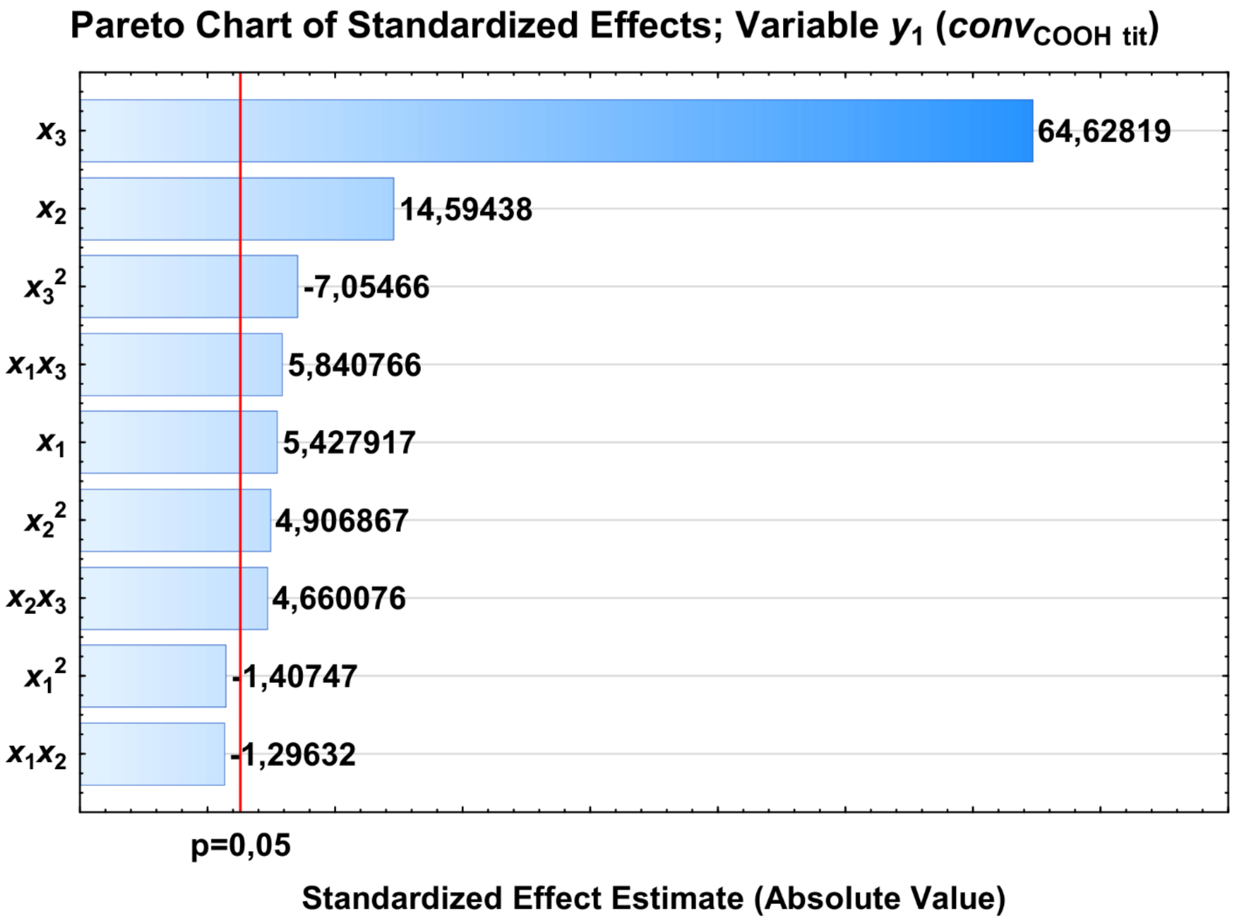

Figure S2. Pareto Chart of Standardize Effects for the $conv_{COOH\ tit}$ (y_1) variable (the red line refers to the limit, beyond which the coefficient of the regression equation becomes significant).

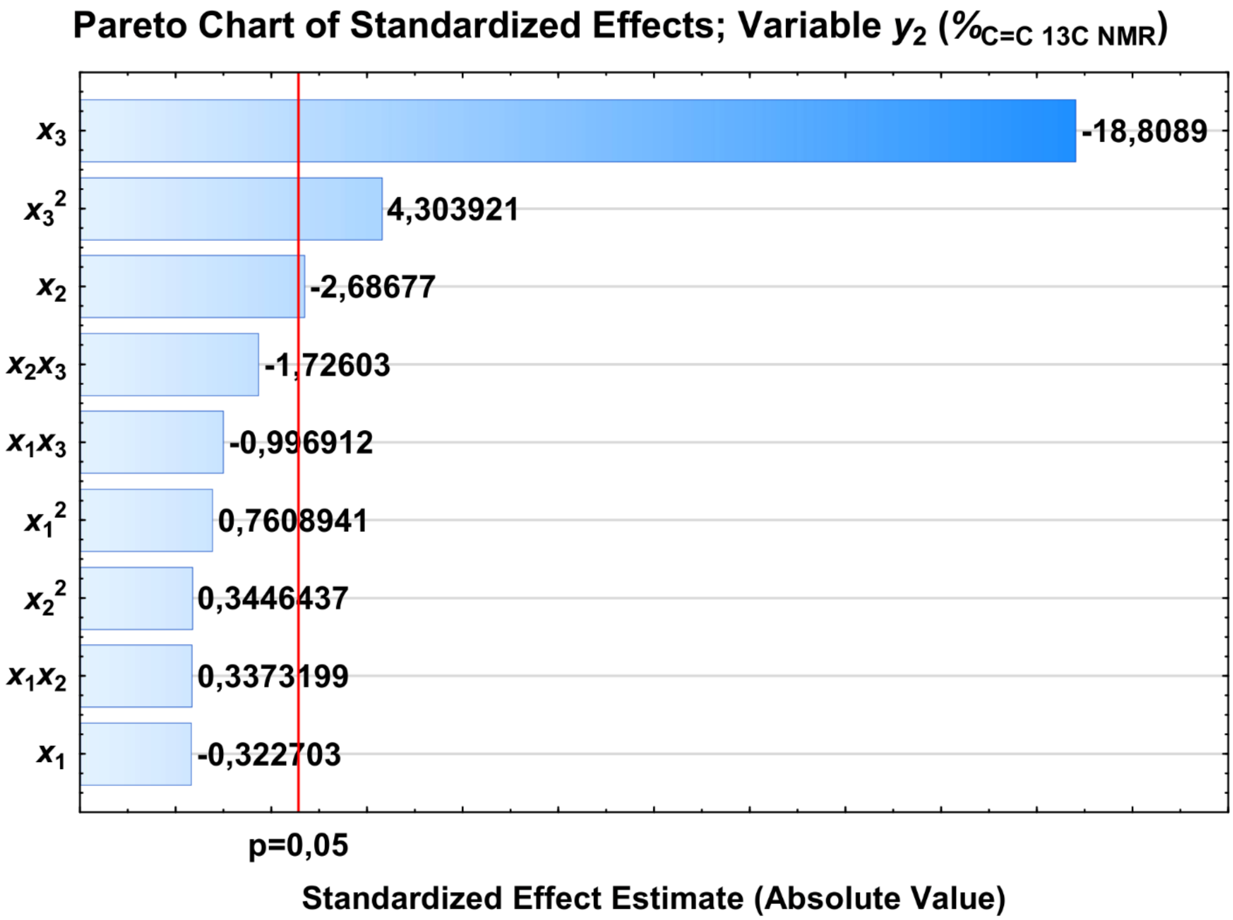

Figure S3. Pareto Chart of Standardize Effects for the $\%_{C=C\ 13C\ NMR}$ (y_2) variable.

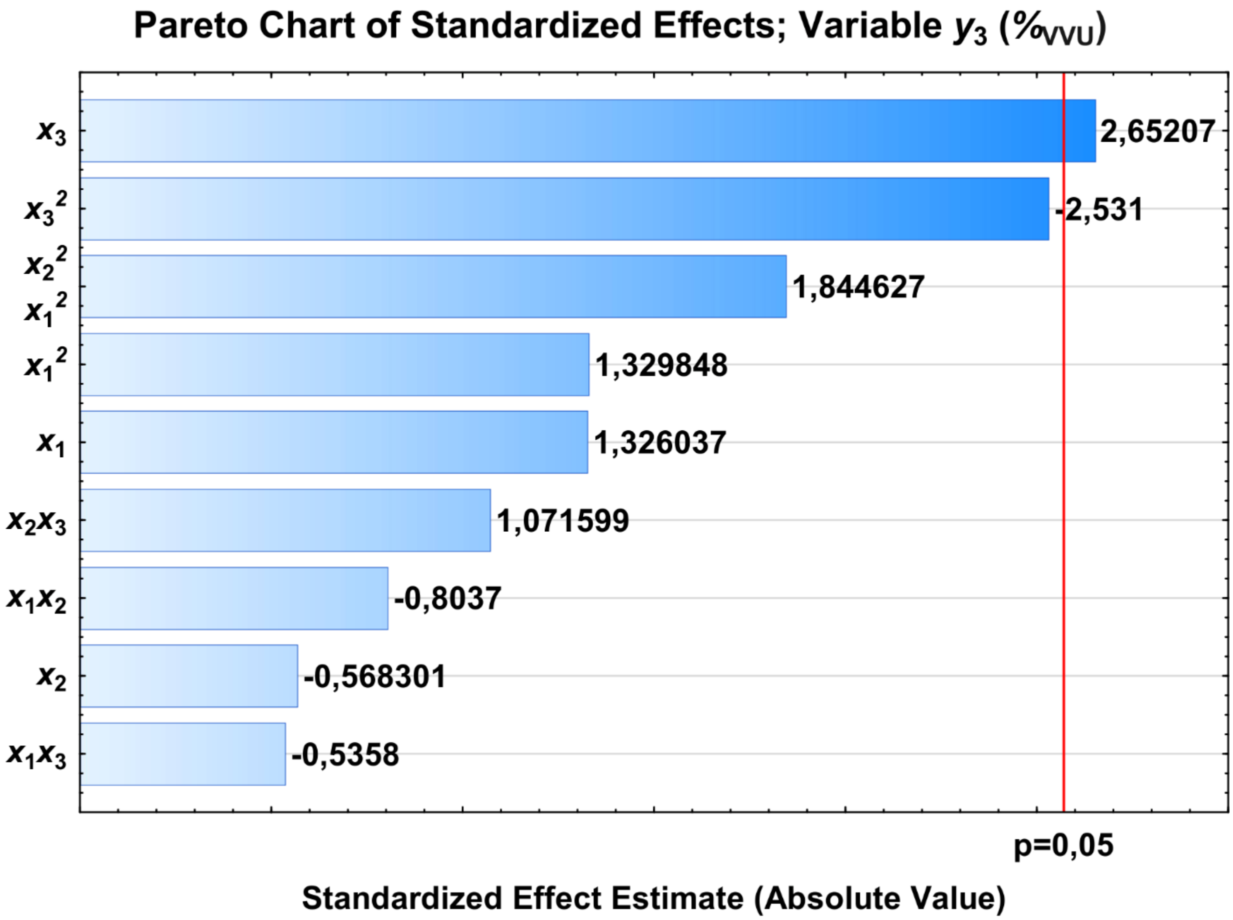

Figure S4. Pareto Chart of Standardize Effects for the $\%_{VVU}$ (y_3) variable.

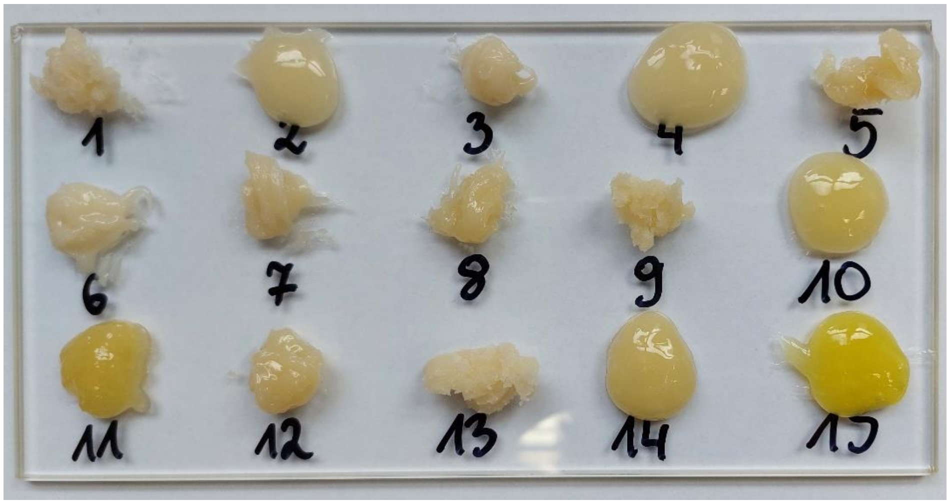

Figure S5. Consistency of the products obtained in the optimization process.

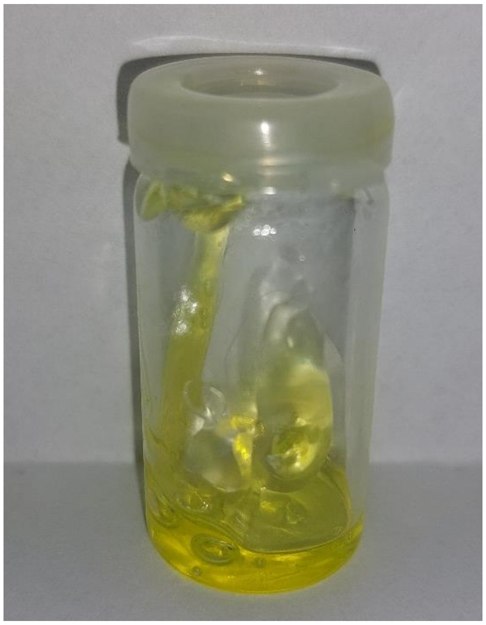

Figure S6. Consistency of the optimal product.

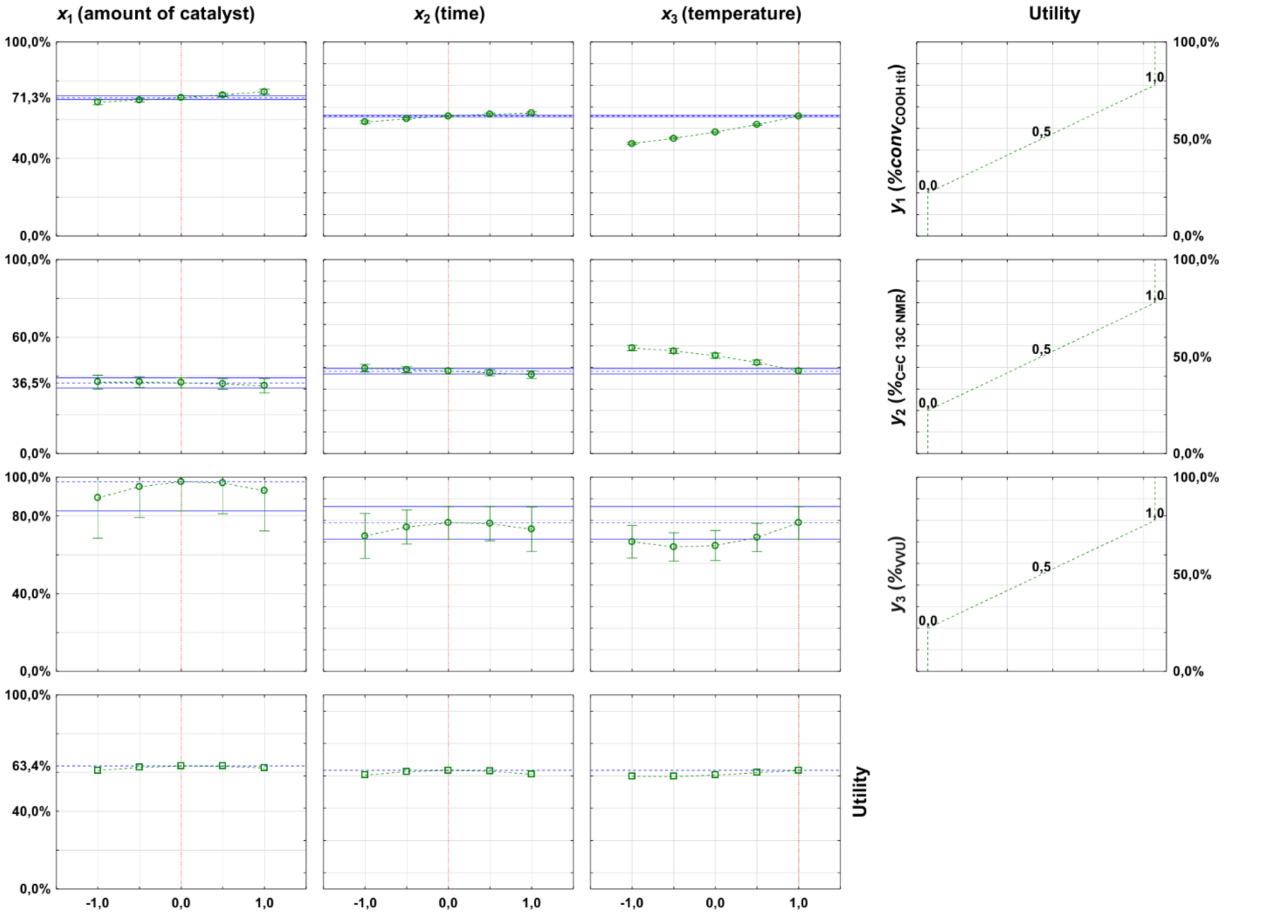

Figure S7. Profile of approximated values of input variables and utility of the used mathematical model.

Table S1. Coded input variables in the Box-Behnken plan for the PBItc synthesis optimization.

Input variable	Value of the coded input variable			Step of the variable
	**-1**	**0**	**1**	
x_1	0.2	0.3	0.4	**0.1**
x_2	3	4	5	**1**
x_3	120	135	150	**15**

Table S2. Number *(*M_n) and weight (M_w) average molar mass and dispersity index (*DI*) calculated from GPC analyses; isomerization to mesaconic acid ($\%Iz_{Mes}$), number average molar weight *(*M_n), and esterification degree (ED_{NMR}) calculated from ^{1}H NMR analyses; percentage conversion of carboxyl groups -COOH calculated from ^{13}C NMR analysis ($conv_{COOH\ 13C\ NMR}$); esterification degree (ED_{tit}), percentage of unreacted unsaturated C=C bonds ($\%_{C=C\ IN}$) calculated from titration analyses and viscosity for the products from the Box-Behnken plan.

No.	Coded variable			GPC Analysis			¹H NMR Analysis			¹³C NMR Analysis	Titration Analysis		*η* [Pa·s]	
	x_1	x_2	x_3	M_n	M_w	*DI*	$\%Iz_{Mes}$ [%]	M_n	ED_{NMR} [%]	$conv_{COOH\ 13C\ NMR}$ [%]	ED_{tit} [%]	$\%_{C=C\ IN}$ [%]	**25°C**	**36,6°C**
**1**	**-1**	**-1**	**0**	791	823	1.04	0.0	865	74.1	54.2	57.8	33.0	2111.1	368.9
**2**	**1**	**-1**	**0**	767	803	1.05	1.5	1327	71.9	54.6	50.4	21.7	40.7	19.4
**3**	**-1**	**1**	**0**	968	1002	1.04	1.9	1420	75.2	62.4	62.9	43.4	1137.0	231.0
**4**	**1**	**1**	**0**	990	1027	1.04	1.8	1618	74.1	64.4	62.6	28.7	810.4	239.3
**5**	**-1**	**0**	**-1**	485	504	1.04	2.0	1005	68.0	48.5	45.7	32.4	73.2	15.5
**6**	**1**	**0**	**-1**	523	541	1.04	1.9	916	65.8	48.1	48.5	34.4	95.2	33.8
**7**	**-1**	**0**	**1**	1275	1347	1.06	2.0	1680	73.4	72.5	71.2	39.9	55.9	18.1
**8**	**1**	**0**	**1**	1345	1461	1.09	2.0	1211	73.1	78.6	78.4	40.2	95.3	33.3
**9**	**0**	**-1**	**-1**	503	515	1.02	0.2	860	65.7	41.6	47.5	28.4	64.1	43.5
**10**	**0**	**1**	**-1**	526	548	1.04	1.9	1215	68.7	50.4	69.9	41.4	184.1	33.5
**11**	**0**	**-1**	**1**	1048	1107	1.06	1.9	1052	72.5	70.2	72.4	33.6	179.0	48.8
**12**	**0**	**1**	**1**	1414	1553	1.10	2.0	1497	75.4	80.0	79.5	35.0	94.0	40.9
**13**	**0**	**0**	**0**	758	789	1.04	2.0	1049	74.5	59.2	57.7	36.2	80.6	45.5
**14**	**0**	**0**	**0**	742	767	1.03	2.0	1132	70.2	60.4	56.2	40.2	205.6	73.7
**15**	**0**	**0**	**0**	718	750	1.05	1.9	1110	68.9	58.6	56.2	38.8	680.6	69.1

Table S3: Values of output variables to generate the response utility profile.

Utility	Output variable		
	$conv_{COOH\ tit}$ [%]	$\%_{C=C\ 13C\ NMR}$ [%]	$\%_{VVU}$ [%]

**Low**	0.0	0.0	0.0
**Medium**	50.0	50.0	50.0
**High**	100.0	100.0	100.0

Table S4. Significance test of regression equation coefficients for the investigated output variable.

Output variable	Input variables/ Parameter relation	Regression coefficient	Standard error	t(5)	p	-95.00% Confidence limit	+95.00% Confidence limit
y_1	Constant	0.565246	0.003213	175.9480	0.000000	0.556987	0.573504
	x_1	0.010678	0.001967	5.4279	0.002877	0.005621	0.015735
	x_1^2	0.004076	0.002896	1.4075	0.218312	-0.003368	0.011520
	x_2	0.028711	0.001967	14.5944	0.000027	0.023654	0.033768
	x_2^2	-0.014209	0.002896	-4.9069	0.004448	-0.021653	-0.006765
	x_3	0.127142	0.001967	64.6282	0.000000	0.122085	0.132200
	x_3^2	0.020429	0.002896	7.0547	0.000884	0.012985	0.027873
	x_1x_2	-0.003607	0.002782	-1.2963	0.251470	-0.010758	0.003545
	x_1x_3	0.016250	0.002782	5.8408	0.002082	0.009098	0.023402
	x_2x_3	0.012965	0.002782	4.6601	0.005532	0.005813	0.020117
y_2	Constant	0,509012	0,009324	54,5935	0,000000	0,485044	0,532979
	x_1	-0,001842	0,005710	-0,3227	0,759978	-0,016519	0,012834
	x_1^2	-0,006395	0,008404	-0,7609	0,481044	-0,027999	0,015209
	x_2	-0,015340	0,005710	-2,6868	0,043466	-0,030017	-0,000663
	x_2^2	-0,002896	0,008404	-0,3446	0,744385	-0,024500	0,018707
	x_3	-0,107391	0,005710	-18,8089	0,000008	-0,122068	-0,092714
	x_3^2	-0,036171	0,008404	-4,3039	0,007687	-0,057775	-0,014567
	x_1x_2	0,002724	0,008075	0,3373	0,749575	-0,018033	0,023480
	x_1x_3	-0,008050	0,008075	-0,9969	0,364577	-0,028806	0,012707
	x_2x_3	-0,013937	0,008075	-1,7260	0,144931	-0,034693	0,006819
y_3	Constant	0,766667	0,053877	14,22983	0,000031	0,628170	0,905163
	x_1	0,043750	0,032993	1,32604	0,242173	-0,041061	0,128561
	x_1^2	-0,064583	0,048564	-1,32985	0,241004	-0,189422	0,060256
	x_2	-0,018750	0,032993	-0,56830	0,594402	-0,103561	0,066061
	x_2^2	-0,089583	0,048564	-1,84463	0,124405	-0,214422	0,035256
	x_3	0,087500	0,032993	2,65207	0,045313	0,002689	0,172311
	x_3^2	0,122917	0,048564	2,53100	0,052466	-0,001922	0,247756
	x_1x_2	-0,037500	0,046659	-0,80370	0,458060	-0,157441	0,082441
	x_1x_3	-0,025000	0,046659	-0,53580	0,615064	-0,144941	0,094941
	x_2x_3	0,050000	0,046659	1,07160	0,332876	-0,069941	0,169941

Table S5. Model adequacy test for the investigated output variables.

	ANOVA – Analysis Of Variance						
Output variable	Source of variation	Sum of squares	Number of degree of freedom	Mean square	$F_{calculated}$	$F_{critical}$	Conclusion
y_1	Regression	1411.24	9	156.80	627.96	19.38	Relevant
	Linear components	1368.29	3	456.10	1826.53	19.16	Relevant
	Non-linear components	42.96	6	7.16	28.67	19.33	Relevant
	Error	1.55	5	-	-	-	-
	Relevance error	1.05	3	0.35	1.40	19.16	**Adequate**

	Random error	0.50	2	0.25	-	-	-
	∑	1412.79	14	-	-	-	-
y_2	Regression	1001.17	9	111.24			
	Linear components	941.72	3	313.91	24.89	19.38	Relevant
	Non-linear components	59.45	6	9.91	70.25	19.16	Relevant
	Error	13.04	5	-	2.22	19.33	Irrelevant
	Relevance error	4.10	3	1.37	-	-	-
	Random error	8.94	2	4.47	0.31	19.16	**Adequate**
	∑	1014.21	14	-	-	-	-
y_3	Regression	2064.58	9	229.40	27.53	19.38	Relevant
	Linear components	793.75	3	264.58	31.75	19.16	Relevant
	Non-linear components	1270.83	6	211.81	25.42	19.33	Relevant
	Error	435.42	5	-	-	-	-
	Relevance error	418.75	3	139.58	16.75	19.16	**Adequate**
	Random error	16.67	2	8.33	-	-	-
	∑	2500.00	14	-	-	-	-

EQUATIONS SECTION:

The following formula was used to calculate the esterification degree (ED_{NMR}) using ^{1}H NMR spectra.

Eq.S1. $ED_{NMR} = ((e'_1 + e'_2) / ((h / 2) + e'_1 + e_1 + e'_2 + e_2 + e'_{O1} + e'_{O2} + g'_O + e'_R)) \times 100\%$

The following formula was used to calculate the isomerization to mesaconic compound contribution ($\%Iz_{Mes}$) using ^{1}H NMR spectra:

Eq.S2. $\%Iz_{Mes} = ((h / 2) / ((h / 2) + e'_1 + e_1 + e'_2 + e_2 + e'_{O1} + e'_{O2} + g'_O + e'_R)) \times 100\%$

The following formula was used to calculate the number of average molar weight (M_n) using ^{1}H NMR spectra:

Eq.S3. $$n = e'_1 + e'_2 + ¼ \times a' + ¼ \times b' + + ½ \times d'$$

Eq.S4. $$M_n = n \times 184.19$$

Where

n – number of repeatable units;

184.19 – the mass of the repeating unit of poly(1,4-butanediol itaconate).

The following formula was used to calculate the carboxyl group conversion ($conv_{COOH\ 13C\ NMR}$) using ^{13}C NMR spectra:

Eq.S5. $$conv_{COOH\ 13C\ NMR} = ((F'_1 + F'_2) / (F'_1 + F'_2 + F_1 + F_2 + I_1 + I_2)) \times 100\%$$

The following formula was used to calculate the Ordelt saturation contribution ($\%Ord$) using ^{13}C NMR spectra:

Eq.S6. $$\%Ord = ((E'_O + G'_O) / (E'_O + G'_O + E'_R + G'_R + E' + G' + E + G)) \times 100\%$$

The following formula was used to calculate the radical polymerization contribution ($\%RP$) using ^{13}C NMR spectra:

Eq.S7. $\%RP = ((E'_R + G'_R) / (E'_O + G'_O + E'_R + G'_R + E' + G' + E + G)) \times 100\%$

The following formula was used to calculate the contribution of unreacted C=C bonds in the reaction system:

Eq.S8. $\%_{C=C} = 100\% - (\%Ord + \%RP)$

The whole regression equation which describes the y_1 variable is:

Eq.S9. $y_1 = 56.5 + 1.07 \times x_1 + 2.87 \times x_2 + 12.7 \times x_3 - 0.361 \times x_1 \times x_2 + 1.63 \times x_1 \times x_3 + 1.30 \times x_2 \times x_3 + 0.408 \times x_1^2 - 1.43 \times x_2^2 + 2.04 \times x_3^2$

The whole regression equation which describes the y_2 variable is:

Eq.S10. $y_2 = 50.9 - 0.184 \times x_1 - 1.53 \times x_2 - 10.7 \times x_3 + 0.272 \times x_1 \times x_2 - 0.805 \times x_1 \times x_3 - 1.39 \times x_2 \times x_3 - 0.640 \times x_1^2 - 0.290 \times x_2^2 - 3.62 \times x_3^2$

The whole regression equation which describes the y_3 variable is:

Eq.S11. $y_3 = 76.7 + 4.37 \times x_1 - 1.88 \times x_2 + 8.75 \times x_3 - 3.75 \times x_1 \times x_2 - 2.50 \times x_1 \times x_3 + 5.00 \times x_2 \times x_3 - 6.46 \times x_1^2 - 8.96 \times x_2^2 + 12.3 \times x_3^2$

The following formula was used to calculate the statistical heat resistance value:

Eq.S12. $T_s = 0.49 \times [T_{d5\%} + 0.6 \times (T_{d30\%} - T_{d5\%})]$

Where

$T_{d5\%}$ - 5% decomposition temperature;

$T_{d30\%}$ - 30% decomposition temperature.
